# Liver-derived ceramides link metabolism to tissue wasting in cancer cachexia

**DOI:** 10.1172/JCI206031

**Published:** 2026-05-15

**Authors:** Kerui Huang, Norbert Perrimon, Marcus D. Goncalves

**Affiliations:** 1 Department of Genetics, Blavatnik Institute, Harvard Medical School, Harvard University, Boston, Massachusetts, USA.; 2 Howard Hughes Medical Institute, Boston, Massachusetts, USA.; 3 NYU Grossman School of Medicine, New York, New York, USA.

## Abstract

Cancer cachexia, characterized by weight loss, muscle wasting, and anorexia, complicates cancer treatment and adversely affects patient outcomes. Both tumor-derived and host inflammatory factors are implicated in aspects of cachexia. The search for circulating mediators of cancer cachexia has focused largely on secreted proteins, but metabolites may also drive systemic wasting. In this issue, Morigny, Rohm, and colleagues identified the liver as a major source of circulating ceramides in cachectic mice and patients with cancer and demonstrated that inhibiting ceramide synthesis attenuated muscle wasting and preserved function in cachectic mice. These findings position the liver as an endocrine organ in cachexia and introduce a druggable metabolic pathway with translational potential.

## Circulating metabolite signals in cachexia

Cancer cachexia is a systemic metabolic syndrome characterized by unintentional weight loss, skeletal muscle atrophy, and reduced physical function (1). The mechanisms driving tissue wasting are incompletely understood, but tumor-secreted factors and host inflammatory responses activate catabolic pathways and suppress anabolic signaling across multiple organs. To date, the search for cachexia-promoting mediators has focused primarily on secreted proteins: cytokines such as IL-6, tumor-derived factors including GDF15 and activin A, and neuropeptides that suppress appetite have all been implicated in specific aspects of the syndrome (1). Yet the extent to which circulating metabolites serve as direct effectors of tissue wasting remains largely unexplored. This question is particularly relevant since cachexia is characterized by increased lipolysis and inflammation, which reshape circulating lipid signals and their effects on peripheral tissues.

Ceramides are a family of bioactive sphingolipids that have emerged as circulating signals linking metabolic dysfunction to end-organ damage (2). Their de novo synthesis is initiated by serine palmitoyltransferase (SPT), the rate-limiting enzyme, and is driven by inflammatory signals and saturated fatty acid flux (2, 3). Once synthesized, ceramides can be released from the liver and circulate to peripheral tissues, where they disrupt mitochondrial integrity and impair oxidative phosphorylation (3–6). Elevated circulating ceramides are associated with obesity, type 2 diabetes, and cardiovascular disease, where they serve as both biomarkers and putative effectors (2, 7). Whether ceramides act as causal mediators in cachexia, however, has remained unclear.

## Targeting hepatic ceramide synthesis preserves muscle mass

In this issue of the *JCI*, Morigny et al. addressed this question using the C26 colon carcinoma model of cachexia (8), which exhibits elevated levels of circulating ceramides. The authors first established the liver as the dominant source of these circulating ceramides in cachectic mice: hepatocyte-specific knockdown of the SPT subunit SPTLC2 via adeno-associated virus–delivered (AAV-delivered) miRNA (miR) nearly completely normalized hepatic and plasma ceramide levels. Having identified the tissue of origin, they then tested the therapeutic relevance of this pathway. Pharmacologic inhibition of SPT with myriocin significantly reduced body weight loss and preserved cardiac and skeletal muscle mass and grip strength, all without affecting tumor growth in the cachectic mice. Adipose tissue loss, by contrast, was unaffected. Hepatocyte-specific genetic knockdown of SPTLC2 recapitulated these effects, confirming that the benefits were attributable to ceramide synthesis rather than off-target activity of myriocin. The concordance between pharmacologic and genetic approaches, tested across early and advanced cachexia time points, substantially strengthens the causal inference.

**Figure 1:**
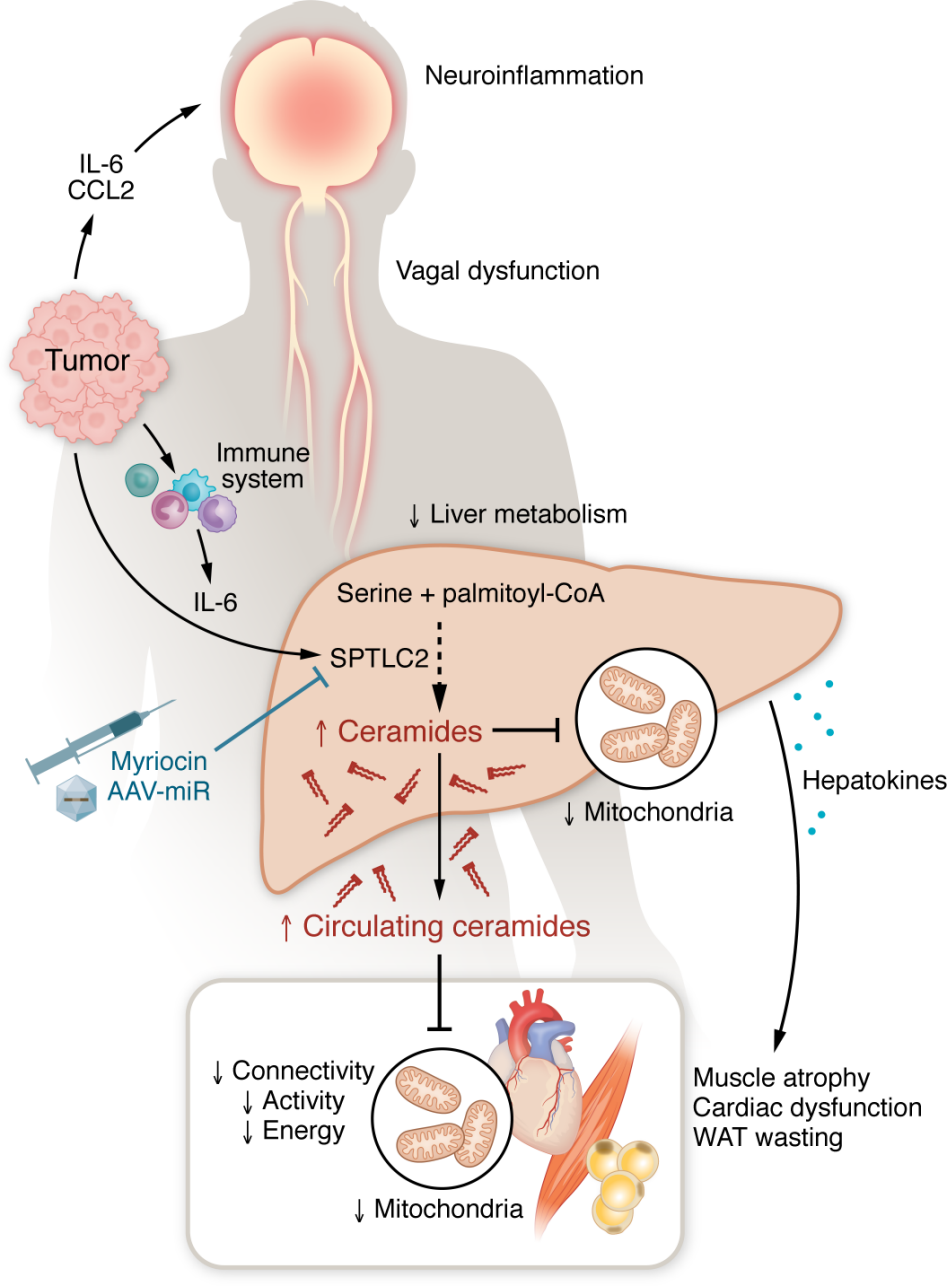
Elevated hepatic production of ceramides drive energetic failure in cachexia. Schematic illustration depicts the liver as a central hub that integrates tumor-, immune-, and CNS-derived signals to orchestrate cachexia-associated metabolic rewiring, producing lipotoxic mediators and hepatokines that drive muscle atrophy, cardiac dysfunction, and white adipose tissue (WAT) wasting. The findings of Morigny, Rohm, and colleagues (8) implicate increased circulating ceramides in cachexia’s complex pathobiology and position sphingolipid metabolism as a potential target for cachexia interventions.

A critical advance in this study is the dissociation of hepatic ceramide synthesis from lipolysis-driven fatty acid flux. Animals subjected to 24-hour fasting, which recapitulates the body composition changes and elevated circulating fatty acids seen in cachexia, showed no increase in hepatic ceramide synthesis enzyme expression. Instead, IL-6 neutralization in C26 tumor–bearing mice almost completely reversed the upregulation of these enzymes, and LPS challenge reproduced the induction. These experiments place inflammatory signaling, not substrate availability, as the upstream trigger. An emerging body of work places the liver at the center of cachexia pathogenesis, whereby inflammation, neural inputs, and intrinsic transcriptional programs converge to drive the production of circulating factors that promote systemic wasting (9, 10). The current study extends this framework by demonstrating that the liver converts tumor-driven inflammation into a circulating lipotoxic signal (Figure 1).

## Elevated ceramides drive energetic failure by impairing mitochondria

Morigny, Rohm, and colleagues next showed that the mechanistic link between ceramide synthesis and muscle wasting runs through mitochondria (8). Liver proteomics identified mitochondrial dysfunction as the top pathway affected by cachexia and rescued by myriocin. Ceramide content in crude liver mitochondria increased 2-fold in cachectic mice, with the ceramide species CER(16:0) accumulating 4-fold. This species, produced primarily by ceramide synthase 6 (CerS6), promotes mitochondrial fission and respiratory dysfunction in other metabolic disease contexts (11). Confocal and electron microscopy confirmed reduced mitochondrial connectivity and altered morphology in cachectic livers, with rescue by myriocin. Similar defects were observed in skeletal muscle and were partially reversed by both pharmacologic and liver-specific genetic inhibition. In vitro, treatment of primary hepatocytes and C2C12 myotubes with CER(16:0) reduced respiration and ATP production and induced dose-dependent myotube atrophy. These data support a model in which liver-derived ceramides impair mitochondrial function locally and in distant tissues, contributing to the energetic failure that underlies wasting.

Notably, myriocin treatment did not alter fat mass. This tissue-specific response suggests differential requirements for ceramide across metabolic tissues and underscores the need to consider cachexia as a composite syndrome with independently targetable components. Human data presented in this study provide translational grounding: In 37 patients with gastrointestinal cancers stratified by the Fearon weight loss criteria, hepatic expression of ceramide synthesis enzymes was elevated in severe cachexia and correlated with the severity of weight loss. This upregulation was liver specific; no comparable changes occurred in adipose tissue or muscle. Hepatic ceramide levels mirrored the species profile seen in mice, with CER(16:0), CER(18:0), and CER(24:1) most prominently increased.

## Outstanding questions and translational implications

Several questions remain. First, ceramides appear to act in parallel with, rather than upstream of, other cachectic drivers. Myriocin did not affect food intake, and effects on energy expenditure were not detected at early cachexia stages. Plasma IL-6 levels were reduced only at advanced time points, and acute-phase response markers were unaffected. This suggests that targeting ceramides would primarily improve tissue resilience to metabolic stress rather than correct anorexia or systemic inflammation, a distinction with practical implications for combination therapy.

Second, myriocin inhibited de novo synthesis at the first committed step, reducing not only ceramides but also hexosyl-ceramides, lactosyl-ceramides, and sphingomyelin. Therefore, it is difficult to attribute the observed phenotypes to a single lipid species. CerS isoform–specific interventions and in vivo metabolic flux measurements will be needed to define the hierarchy among sphingolipid mediators. The route of inter-organ delivery also warrants investigation. Ceramides circulate in association with lipoproteins and other carriers (2), but the relative contribution of these pathways in cachexia is unknown. Notably, liver-specific SPTLC2 knockdown normalized CER(16:0) in muscle without affecting CER(18:0) or CER(18:1), the predominant locally synthesized species. This partial independence of hepatic and muscle ceramide pools suggests distinct origins and potentially distinct functions.

Despite these uncertainties, the translational implications are substantial. Even partial preservation of skeletal muscle mass during cancer treatment is associated with improvements in quality of life, delayed disease progression, and improved survival (12–14). Several agents modulating sphingolipid metabolism are in development, including CerS inhibitors, the DEGS1 inhibitor fenretinide, and sphingosine kinase inhibitors already in early-phase clinical trials (15). Circulating ceramide species may also serve as biomarkers to identify patients at risk for cachexia before clinical weight loss becomes apparent.

More broadly, these findings challenge the expectation that cachexia will yield to a single unifying mechanism. The field has often sought one pathway to explain the entire phenotype, but this assumption may obscure tractable biology. Cachexia is more appropriately viewed as a composite syndrome in which inflammation, anorexia, and metabolic dysfunction interact but can be individually targeted. Morigny, Rohm, and colleagues demonstrate that meaningful progress comes from isolating specific components. Ceramides define a liver-to-periphery metabolic axis that is biologically coherent, supported by human data, and amenable to pharmacologic intervention. The next step is to determine whether sphingolipid-targeted therapies can be combined with nutritional support, antiinflammatory agents, or standard anticancer treatments to achieve meaningful clinical benefit in patients with cancer cachexia.

## Conflict of interest

MDG reports equity ownership in Sensei Biotherapeutics, has received consulting fees from Genentech, and is an inventor on patent applications related to cancer metabolism and cachexia (WO2025189122A1, WO2020191356A1, WO2023070023A1, US20260014164A1).

## Funding support

This work was supported by NIH funding and is subject to the NIH Public Access Policy. Through acceptance of this funding, the NIH has been granted a right to make the work publicly available in PubMed Central.

- This work was also delivered as part of the CANCAN team supported by the Cancer Grand Challenges partnership funded by Cancer Research UK (CGCATF-2021/100022) and the National Cancer Institute (NCI), NIH (OT2 CA278685-01).

## Version 1 05/15/2026

Electronic publication
